# Fluoride mitigates aluminum-toxicity in barley: morpho-physiological responses and biochemical mechanisms

**DOI:** 10.1186/s12870-022-03610-z

**Published:** 2022-06-13

**Authors:** Mona F. A. Dawood, Md. Tahjib-Ul-Arif, Abdullah Al Mamun Sohag, Arafat Abdel Hamed Abdel Latef

**Affiliations:** 1 Botany and Microbiology Department, Faculty of Science, Assiut University, Assiut, 71516 Egypt; 2 Department of Biochemistry and Molecular Biology, Faculty of Agriculture, Bangladesh Agricultural University, Mymensingh, 2202 Bangladesh; 3 Botany and Microbiology Department, Faculty of Science, South Valley University, Qena, 83523 Egypt

**Keywords:** Aluminum chloride, Antioxidant enzymes, Barley, Heavy metal stress, Oxidative stress

## Abstract

**Background:**

To our knowledge, the role of exogenous fluoride (F^–^) on aluminum (Al)-stress mitigation in plants has not been investigated yet. In this experiment, barley (*Hordeum vulgaris*) seedlings were exposed to excessive Al^3+^ concentrations (aluminum chloride, 0.5, 1.0, 2.0, 3.0, and 4.0 mM) with and without fluoride (0.025% sodium fluoride) to explore the possible roles of fluoride on the alleviation of Al-toxicity.

**Results:**

Overall, Al-stress caused inhibition of growth and the production of photosynthetic pigments. Principal component analysis showed that the growth inhibitory effects were driven by increased oxidative stress and the interruption of water balance in barley under Al-stress. Fluoride priming, on the other hand, enhanced growth traits, chlorophyll *a* and *b* content, as well as invigorated the protection against oxidative damage by enhancing overall antioxidant capacity. Fluoride also improved osmotic balance by protecting the plasma membrane. Fluoride reduced endogenous Al^3+^ content, restored Al-induced inhibition of glutathione-S-transferase, and increased  the contents of phytochelatins and metallothioneins, suggesting that fluoride reduced Al^3+^ uptake and improved chelation of Al^3+^.

**Conclusions:**

Aluminum chloride-induced harmful effects are abridged by sodium fluoride on barely via enhancing antioxidative responses, the chelation mechanism causing reduction of Al uptake and accumulation of barely tissues. Advanced investigations are necessary to uncover the putative mechanisms underpinning fluoride-induced Al-stress tolerance in barley and other economically significant crops, where our results might serve as a solid reference.

**Supplementary Information:**

The online version contains supplementary material available at 10.1186/s12870-022-03610-z.

## Background

Soil heavy metal contamination is an acute impediment to sustainable crop cultivation. The third most prevalent metal element in the earth's crust is aluminum (Al), denoting nearly 8.1% of its content in weight [1]. Aluminum has no essential function in biological processes; instead, it elicits toxicity in plants when found in an excessive amount in the soil solution [2, 3]. Several factors influence Al-induced toxicity in plants, such as pH of the soil, ionic species of Al, crop genotypes, and growth conditions [1, 2]. Al-stress causes numerous negative impacts, including but not limited to morpho-physiological, biochemical, and molecular alterations in plants, causing stunted growth, delayed developmental processes, and lower productivity of crops [2, 3]. Al-toxicity is most common in acidic soils around the world [2]. Several investigations have highlighted the mechanisms of Al-stress tolerance in several plant species during the last few decades [4–7]. Al is present in the soil as non-toxic chemical forms such as aluminum oxides or aluminosilicates, but a soil solution with an acidic pH triggers the release of various toxic ionic forms of Al, of which Al^3+^ is the most abundant and toxic to plants [8–10]. Estimates have revealed that around 40% of arable lands all over the world are already acidic; thus, any subsequent escalation of soil acidity due to acid rain and anthropogenic activity can further exacerbate the menace of Al-toxicity [10, 11]. Therefore, continuous examination of the toxicological impact of Al in plants is indispensable for developing strategies for mitigating its toxicity.

Previous experiments demonstrated that after penetrating cells, Al^3+^ stimulates the generation of reactive oxygen species (ROS), such as singlet oxygen (^1^O_2_), hydrogen peroxide (H_2_O_2_), superoxide (O_2_^•−^), and hydroxyl radical (^•^OH); these induce oxidative stress to cellular components [6, 12, 13]. The antioxidant systems, enzymatic antioxidants (e.g., superoxide dismutase (SOD), catalase (CAT), glutathione peroxidase (GPX), and ascorbate peroxidase (APX)), and non-enzymatic antioxidants (e.g., reduced glutathione (GSH) and ascorbic acid (ASA)), counteract oxidative damage [2, 6, 13, 14]. The discrepancy between the formation and elimination of ROS impairs plant cell redox equilibrium, resulting in inhibition of functions of biomolecules and the plasma membrane [13]. Furthermore, as a defense mechanism, plants rely on chelation, which occurs when a central metal atom/ion interacts with a ligand and leads to the formation of a complex ring-like structure to detoxify heavy metals, including Al^3+^ [15]. Al-stress additionally interferes with a variety of other biological processes, such as the breakdown of photosynthetic pigments, as a result of which photosynthesis is reduced [16–19], and unbalanced nitrogen metabolism by lowering nitrate reductase (NR) content and nitric oxide (NO) production [20]. Many measures, such as the assortment of Al-stress tolerant cultivars, the production of Al-stress tolerant transgenic lines, and chemical priming have recently been implemented to reduce the detrimental effects of Al on plants. In terms of time and cost, chemical priming has shown to be the most promising of these options. As a result, scientists are working hard to find viable compounds for priming.

Fluoride (F^−^) is a well-known pollutant in the environment for its highly reactive and non-biodegradable nature, which ranks 13^th^ in abundance in the Earth’s crust [21, 22]. Most of the research findings in plants suggest that excessive accumulation of fluoride causes phytotoxicity [21]. However, fluoride at low concentrations is beneficial in the prevention of dental caries and facilitates the mineralization of hard tissues. A recent study demonstrated that fluoride toxicity in tea plants was reduced by aluminum chloride (AlCl_3_) and sodium fluoride (NaF) co-treatment due to the formation of Al-F complexes [22]. Thus, we hypothesized that fluoride at a low dose might reduce the Al-stress as it forms Al-F complexes.

Barley (*Hordeum vulgaris*) is the fourth most widely grown cereal crop worldwide and is vulnerable to Al-stress and acidic soils, causing significant yield loss [23, 24]. However, so far, no studies have specifically addressed, as a priming compound, how fluoride may reduce the Al-toxicity in barley. To address this knowledge gap, we investigated the roles of exogenous fluoride on (i) barley plant growth response, (ii) photosynthetic pigment contents, (iii) water relation-related parameters, (iv) oxidative and metallic stress markers, (v) enzymatic antioxidant activities, and (vi) non-enzymatic antioxidant contents under Al-stress. To the best of our knowledge, this study is the first attempt to explore fluoride-mediated Al-stress mitigation efficiency in barley plants.

## Results

### Effects of fluoride on growth, photosynthetic pigments and water relations under Al-stress

**Fig. 1:**
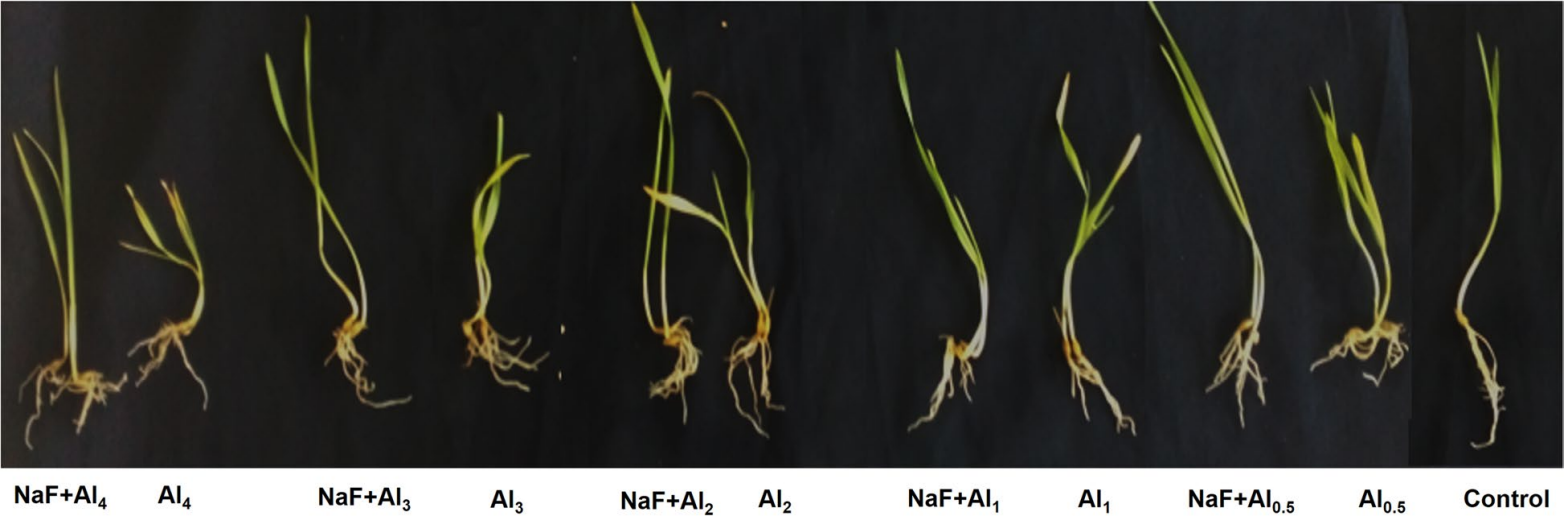
Effects of NaF priming on the phenotypic appearance of barley seedlings were grown with and without the presence of different concentrations of AlCl_3_. ‘Control’, 0 mM AlCl_3_ + 0% NaF; ‘Al_0.5_’, 0.5 mM AlCl_3_ + 0% NaF; ‘NaF+Al_0.5_’, 0.5 mM AlCl_3_ + 0.025% NaF; ‘Al_1_’, 1 mM AlCl_3_ + 0% NaF; ‘NaF+Al_1_’, 1 mM AlCl_3_ + 0.025% NaF; ‘Al_2_’, 2 mM AlCl_3_ + 0% NaF; ‘NaF+Al_2_’, 2 mM AlCl_3_ + 0.025% NaF; ‘Al_3_’, 3 mM AlCl_3_ + 0% NaF; ‘NaF+Al_3_’, 3 mM AlCl_3_ + 0.025% NaF; ‘Al_4_’, 4 mM AlCl_3_ + 0% NaF; ‘NaF+Al_4_’, 4 mM AlCl_3_ + 0.025% NaF

We initially investigated whether exogenous sodium fluoride (NaF) provides tolerance to Al-stress, a harmful metal that severely impairs plant growth characteristics, water relations, and photosynthetic pigments [2]. As expected, shoot length (SL), root length (RL), plant fresh weight (PFW), and plant dry weight (PDW) of barley seedlings were reduced in a dose-dependent manner under Al-stress, with a dramatic drop by 49.10, 63.41, 60.40, and 46.49%, respectively, noted in the 'Al4' treatment relative to the control treatment (Table 1). A comparable outcome was also observed in ryegrass (*Lolium multiflorum*) [25]. Notably, exogenous NaF treatments in all Al treatments significantly increased the SL, RL, PFW, and PDW compared to only Al-stressed barley plants (Table 1). The phenotypic improvements of NaF-treated barley plants were observed in the pictures (Fig. 1). Relative water content (RWC), epicuticular wax content, chlorophyll (Chl) *a*, Chl *b*, and carotenoid contents of barley leaves in response to Al-stress were lowered compared to control condition, in a dose-dependent manner (Table 1). Similar outcomes were also documented in the case of many other species of plants, such as ryegrass and high bush blueberry (*Vaccinium corymbosum*) [25, 26]. However, NaF considerably increased these parameters in Al-stressed barley plants relative to their exclusively Al-stressed seedlings (Table 1). Proline content increased substantially at the root in Al-stressed barley plants relative to control plants (Table 1), as also observed in mung bean (*Vigna radiata*) [27]. Surprisingly, non-significant difference in shoot proline content was seen when only Al-treatments were compared to the control treatment (Table 1). However, NaF treatment on Al-stressed plants significantly reduced root proline content in the 'NaF+Al_1_', 'NaF+Al_2_', 'NaF+Al_3_', and 'NaF+Al_4_' treatments, but no significant change in shoot proline content was seen when compared to their respective only Al-stressed treatments (Table 1). Overall, these findings imply that applying NaF to barley plants can help to reduce Al-toxicity.

**Table 1:** Effects of NaF priming on growth, photosynthetic pigments and water relations under Al-stress in barley plants

Treatments	Shoot length (cm)	Root length (cm)	Plant fresh weight (mg)	Plant dry weight (mg)	Relative Water Content (%)	Epiculticular Wax (mg m^**-2**^)	Chl ***a*** (mg 100g^**-1**^ FW)	Chl ***b*** (mg 100g^**-1**^ FW)	Chl *a*+*b* (mg 100g^**-1**^ FW)	Carotenoid content (mg 100g^**-1**^ FW)	Proline shoot (mg g^**-1**^ FW)	Proline root (mg g^**-1**^ FW)
**C**	12.96±0.2^c^	4.10±0.2^c^	289.50±3.4^b^	27.60±0.4^bc^	86.39±0.5^a^	35.43±1.4^bcd^	10.14±0.4^b^	4.57±0.24^bc^	14.71±0.2^cd^	7.38±0.39^b^	4.24±0.3^ab^	0.53±0.03^e^
**Al0.5**	10.00±00^d^	3.66±0.1^cd^	222.13±6.8^de^	30.00±2.1^b^	80.33±0.5^bc^	31.01±1.3^cde^	8.83±0.3^bcd^	3.85±0.15^d^	12.68±0.5^ef^	5.53±0.22^c^	4.89±0.4^ab^	1.52±0.14cd
**Al1**	10.03±0.1^d^	3.00±00^de^	200.20±0.8^ef^	17.50±0.3^ef^	75.21±0.5^d^	29.28±1.2^def^	8.18±0.3^cd^	3.05±0.16^e^	11.23±0.2^f^	4.50±0.24^cd^	4.84±0.4^ab^	2.39±0.01^b^
**Al2**	9.80±0.1^d^	2.73±0.1^e^	173.00±2.8^fg^	16.90±0.2f	70.33±0.4^e^	28.30±1.2^ef^	6.08±0.2^ef^	2.86±0.11^e^	8.94±0.4^g^	4.17±0.17^de^	4.91±0.4^ab^	3.85±0.19^a^
**Al3**	8.63±0.1^e^	2.40±0.1^e^	166.23±5.4^g^	16.06±0.4^f^	63.86±0.4^f^	26.81±1.1^ef^	5.13±0.2^fg^	2.53±0.10^e^	7.66±0.3^gh^	4.07±0.16^de^	4.99±0.4^ab^	4.23±0.26a
**Al4**	6.60±0.2^f^	1.50±0.1^f^	114.63±6.4^h^	14.76±0.2^f^	56.39±0.3^g^	23.66±1.0^f^	4.48±0.2^g^	1.80±0.07^f^	6.28±0.2^h^	3.09±0.12^e^	5.83±0.4^a^	4.51±0.28^a^
**NaF**	14.03±0.3^b^	6.06±0.2^a^	330.33±3.2^a^	40.00±0.6^a^	86.88±0.5^a^	38.34±1.6^ab^	12.72±0.5^a^	5.46±0.22^a^	18.19±0.7a	8.40±0.34^ab^	3.83±0.3^b^	0.42±0.03^e^
**NaF+Al0.5**	15.23±0.1^a^	4.10±0.1^c^	252.43±7.3^c^	31.06±1.2^b^	86.36±0.5^a^	39.01±1.1^ab^	9.86±0.1^b^	4.96±0.03^ab^	14.82±0.1^cd^	7.88±0.05^b^	4.89±0.4^ab^	0.82±0.01^de^
**NaF+Al1**	15.10±0.2^a^	4.30±0.2^bc^	245.00±4.4^cd^	28.50±0.5^bc^	86.52±0.5^a^	41.30±1.1^ab^	12.01±0.1^a^	4.36±0.02^bcd^	16.37±0.1^bc^	8.19±005^ab^	4.31±0.3^ab^	0.90±0.01^de^
**NaF+Al2**	13.80±0.1b^c^	5.10±0.2^b^	200.20±5.7^ef^	25.46±0.6^cd^	85.39±0.5^a^	42.75±1.2^a^	12.45±0.1^a^	4.47±0.02^bcd^	16.93±0.1^ab^	9.09±0.05^a^	4.89±0.4^ab^	1.35±0.10^cd^
**NaF+Al3**	13.70±0.1b^c^	4.43±0.2^bc^	243.06±6.7^cd^	27.70±0.4^bc^	80.96±0.5^b^	37.33±0.8^abc^	9.23±0.1^bc^	4.23±0.02^cd^	13.60±0.2^de^	9.07±0.05^a^	3.48±0.2^b^	1.86±0.01^bc^
**NaF+Al4**	9.93±0.2^d^	3.06±0.1^de^	159.96±4.1^g^	21.73±0.8^de^	77.63±0.5^cd^	30.62±1.2^de^	7.42±0.04^de^	3.85±0.02^d^	11.28±0.^1f^	5.46±0.03^c^	3.10±0.2^b^	2.10±0.09^bc^

‘C’, Hydro-primed seeds + 0 mM AlCl_3_; ‘Al_0.5_’, Hydro-primed seeds +0.5 mM AlCl_3_; ‘Al_1_’, Hydro-primed seeds + 1 mM AlCl_3_; ‘Al_2_’, Hydro-primed seeds + 2 mM AlCl_3_; ‘Al_3_’, Hydro-primed seeds + 3 mM AlCl_3_; ‘Al_4_’, Hydro-primed seeds + 4 mM AlCl_3_; ‘NaF’, 0.025% NaF-primed seeds + 0 mM AlCl_3_; ‘NaF+Al_0.5_’, 0.025% NaF-primed seeds + 0.5 mM AlCl_3_; ‘NaF+Al_1_’, 0.025% NaF-primed seeds + 1 mM AlCl_3_; ‘NaF+Al_2_’, 0.025% NaF-primed seeds + 2 mM AlCl_3_; ‘NaF+Al_3_’, 0.025% NaF-primed seeds + 3 mM AlCl_3_; ‘NaF+Al_4_’, 0.025% NaF-primed seeds + 4 mM AlCl_3_. Values are means ± standard errors (SEs) (*n* = 5). Bars followed by the same letter are non-significant among the treatments at *P*≤0.05 based on Tukey’s test. Chl, Chlorophyl

### Effects of fluoride against oxidative damage protection under Al-stress

**Fig. 2:**
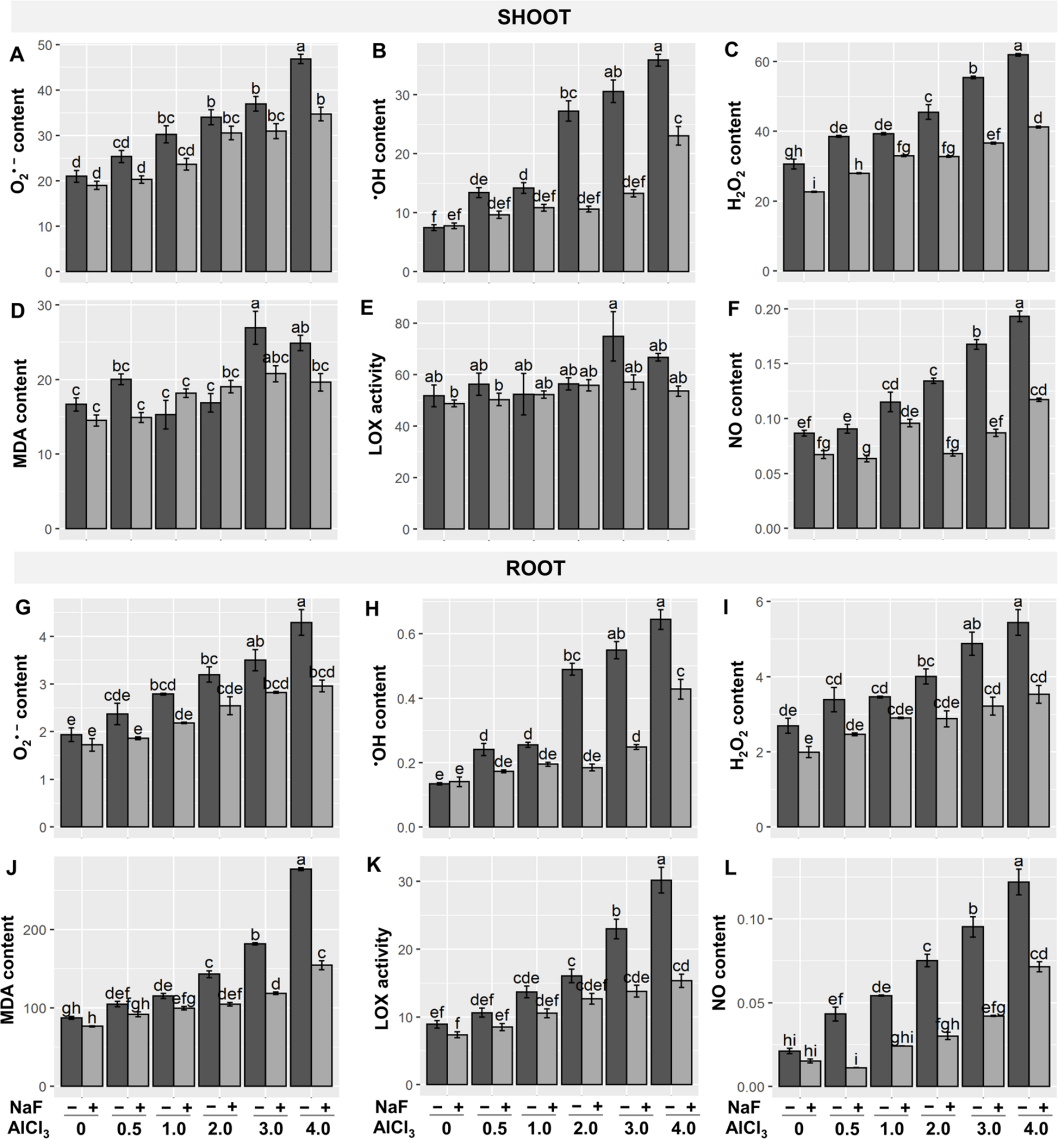
Effects of NaF priming on the superoxide anion (O_2_^•−^) (**A**, **G**), hydroxyl radical (^•^OH) (**B**, **H**), hydrogen peroxide (H_2_O_2_) (**C**, **I**), malondialdehyde (MDA) contents (**D**, **J**), lipoxygenases (LOX) activity (**E**, **K**), and nitric oxide (NO) content (**F**, **L**) in shoots and roots of barley plants under Al-stress. Values are means ± standard errors (SEs) (*n* = 5). According to Tukey's test, bars that are accompanied by the same alphabet are not significant among the treatments at *P*≤0.05

According to a previous study, exposure to excessive Al disturbs cellular redox equilibrium, leading to an overabundance of ROS, which oxidizes biological molecules like lipids, proteins, enzymes, and nucleic acids, eventually causing cell death [28]. As a result, ROS and NO contents were measured to analyze the defensive role of NaF counter to Al-provoked oxidative damage. In comparison to the control treatment, substantial increments of O_2_^•−^, ^•^OH, H_2_O_2_, and malondialdehyde (MDA) contents were observed in Al-stressed treatments in a concentration-dependent fashion (Fig. 2A-D, G-J). Comparable findings have been mentioned in other species of plants [7, 25–27, 29–31]. 'NaF+Al_0.5_', 'NaF+Al_1_', 'NaF+Al_2_', 'NaF+Al_3_', and 'NaF+Al_4_' treatments, on the other hand, considerably lowered the O_2_^•−^, ^•^OH, H_2_O_2_, and MDA contents both at shoots and roots in comparison to their respective only Al-stressed treatments (Fig. 2A-D, G-J) Lipoxygenase (LOX) activity was greatly elevated in the roots of Al-stressed plants by 80.54, 158.49, and 239.24%, respectively, at 'Al_2_', 'Al_3_', and 'Al_4_' treatments, compared to control, as was found in the case of other toxic metals, such as cadmium stress [32]. Interestingly, at the shoot, LOX activity did not differ considerably from control plants (Fig. 2E). However, LOX activity was reduced at the 'NaF+Al_1_', 'NaF+Al_2_', 'NaF+Al_3_', and 'NaF+Al_4_' treatments in the roots (Fig. 2K), but shoot LOX activity did not change significantly (Fig. 2F) when compared to their respective only Al-stressed treatments. The NO content enhanced considerably in a concentration-dependent way at both shoots and roots in Al-stress treatments compared to the control treatment, as shown in our prior investigation under saline-alkaline stress [33]. Exogenous NaF application, on the other hand, reduced NO content in the 'NaF+Al_0.5_', 'NaF+Al_1_', 'NaF+Al_2_', 'NaF+Al_3_', and 'NaF+Al_4_' treatments at both the shoots and roots when compared to their paralleling exclusively Al-exposed plants (Fig. 2F, L).

### Effects of fluoride on antioxidant enzymes activity under Al-stress

**Fig. 3:**
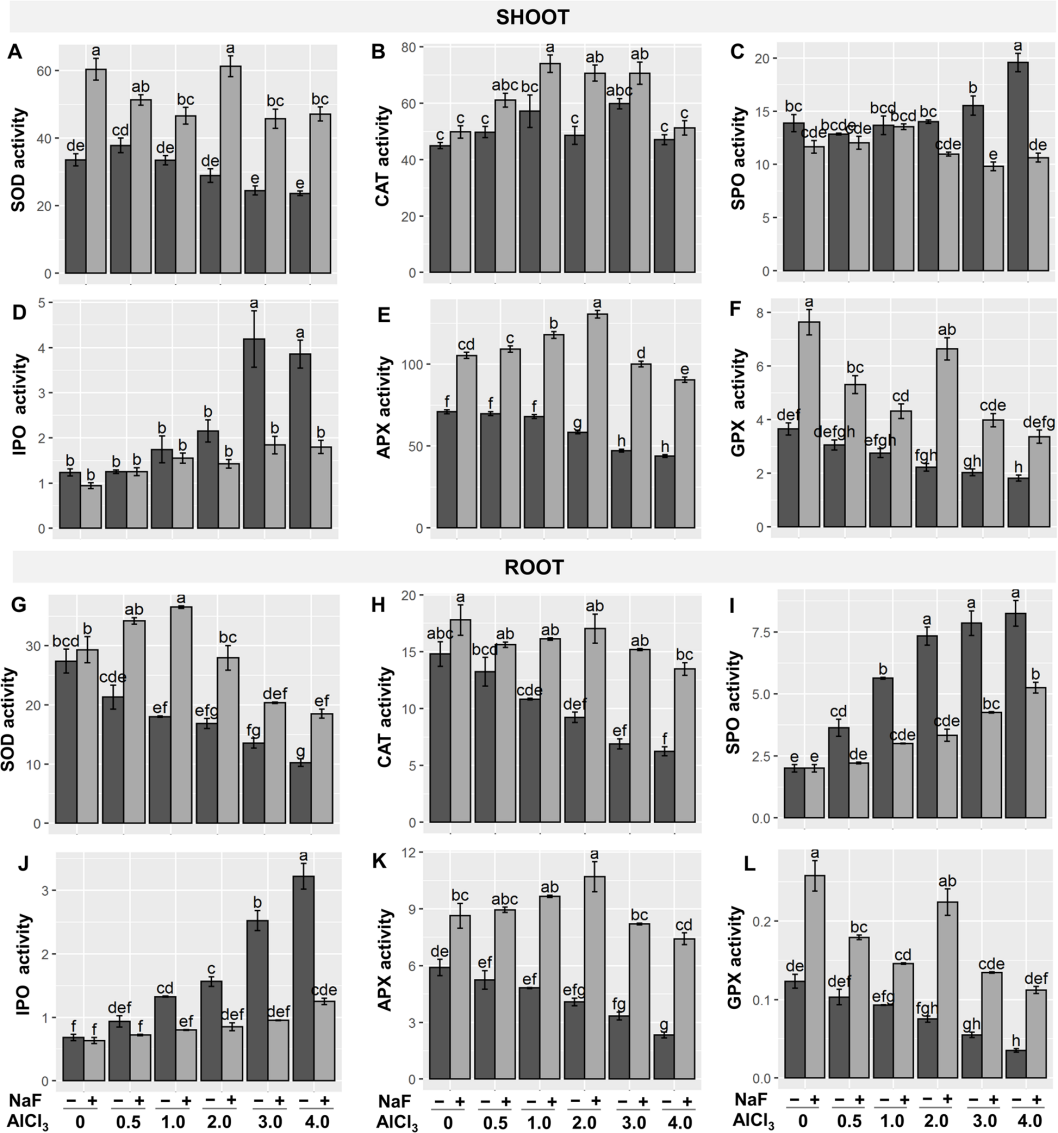
Effects of NaF priming on activities of antioxidant enzymes such as superoxide dismutase (SOD, U mg protein^−1^ g^−1^ FW min^−1^) (**A**, **G**), catalase (CAT, U mg protein^−1^ g^−1^ FW min^−1^) (**B**, **H**), soluble peroxidase (SPO, U mg protein^−1^ min^−1^) (**C**, **I**), ionic peroxidase (IPO, U mg protein^−1^ min^−1^) (**D**, **J**), ascorbate peroxidase (APX, μmol mg protein^−1^ g^−1^ FW min^−1^) (**E**, **K**), and glutathione peroxidase (GPX) (**F**, **L**) in shoots and roots of barley plants under Al-stress. Values are means ± standard errors (SEs) (*n* = 5). According to Tukey's test, bars that are accompanied by the same alphabet are not significant among the treatments at *P*≤0.05

The enzymatic antioxidant system is a key regulator in ROS homeostasis of plants [28]. To comprehend the function of NaF on the activities of some enzymatic antioxidants such as superoxide dismutase (SOD), catalase (CAT), ascorbate peroxidase (APX), soluble peroxidase (SPO), ionic peroxidase (IPO), and glutathione peroxidase (GPX) were studied. SOD activity was considerably reduced in shoot at 'Al_3_' and 'Al_4_' treatments and in root at 'Al_1_', 'Al_2_', 'Al_3_', and 'Al_4_' treatments compared to the control treatment (Fig. 3A, G), which contradicts with the previous results reported in blueberry (*Vaccinium corymbosum*) under Al-stress [26]. On the other hand, exogenous NaF treatment increased both shoot- and root-SOD activities in Al-stressed barley plants when compared to their corresponding exclusively Al-stressed counterparts (Fig. 3A, G).

CAT activity was significantly increased in roots by 37.46, 53.35, and 57.8%, respectively, under 'Al_2_', 'Al_3_', and 'Al_4_' treatments, as also observed in ryegrass [25], but not in shoots as compared to control barley plants (Fig. 3B, H). Contrastingly, exogenous NaF boosted the activity of CAT both in the shoot and the root, except for the 'NaF+Al_4_' treatment in the shoot, relative to their respective solely Al-stressed plants (Fig. 3B, H) The shoot-APX activity was decreased by 17.64, 33.36, 38.05%, respectively, in 'Al_2_', 'Al_3_', and 'Al_4_' treatments and root-APX activity reduced in 'Al_3_' and 'Al_4_' treatments compared to the control treatment (Fig. 3E, K), which is consistent with earlier findings in *Arachis hypogaea* plants [7], but contradict with the results found in *Zea mays* L. (maize) plant [34]. In contrast, NaF treatment significantly boosted shoot-APX and root-APX activities in all Al-stressed plants relative to the paralleling only Al-stressed barley plants (Fig. 3E, K) Under Al-stress, GPX activity reduced in a dose-dependent way at both roots and shoots, with significant decreases reported in 'Al_3_' and 'Al_4_' treatments compared to control (Fig. 3F). However, NaF dramatically increased shoot-GPX and root-GPX activities in Al-stressed plants relative to only Al-stressed plants (Fig. 3F). Under Al-stress, SPO activity was variable at the root but increased in the shoot in a concentration-dependent way relative to the control (Fig. 3C, I). However, NaF application considerably lowered SPO activity at roots in 'NaF+Al_1_', 'NaF+Al_2_', 'NaF+Al_3_', and 'NaF+Al_4_' treatments and at shoots in 'NaF+Al_3_' and 'NaF+Al_4_' treatments when compared to their respective just Al-stressed treatments (Fig 3C, I). Under Al-stress, shoot-IPO and root-IPO activities amplified in a concentration-dependent way when paralleled to control (Fig. 3D, J). When compared to only Al-stressed plants, exogenous NaF treatment significantly reduced IPO activity at both the root and shoot (Fig. 3D, G).

### Effects of NaF on non-enzymatic antioxidants under Al-stress

**Fig. 4:**
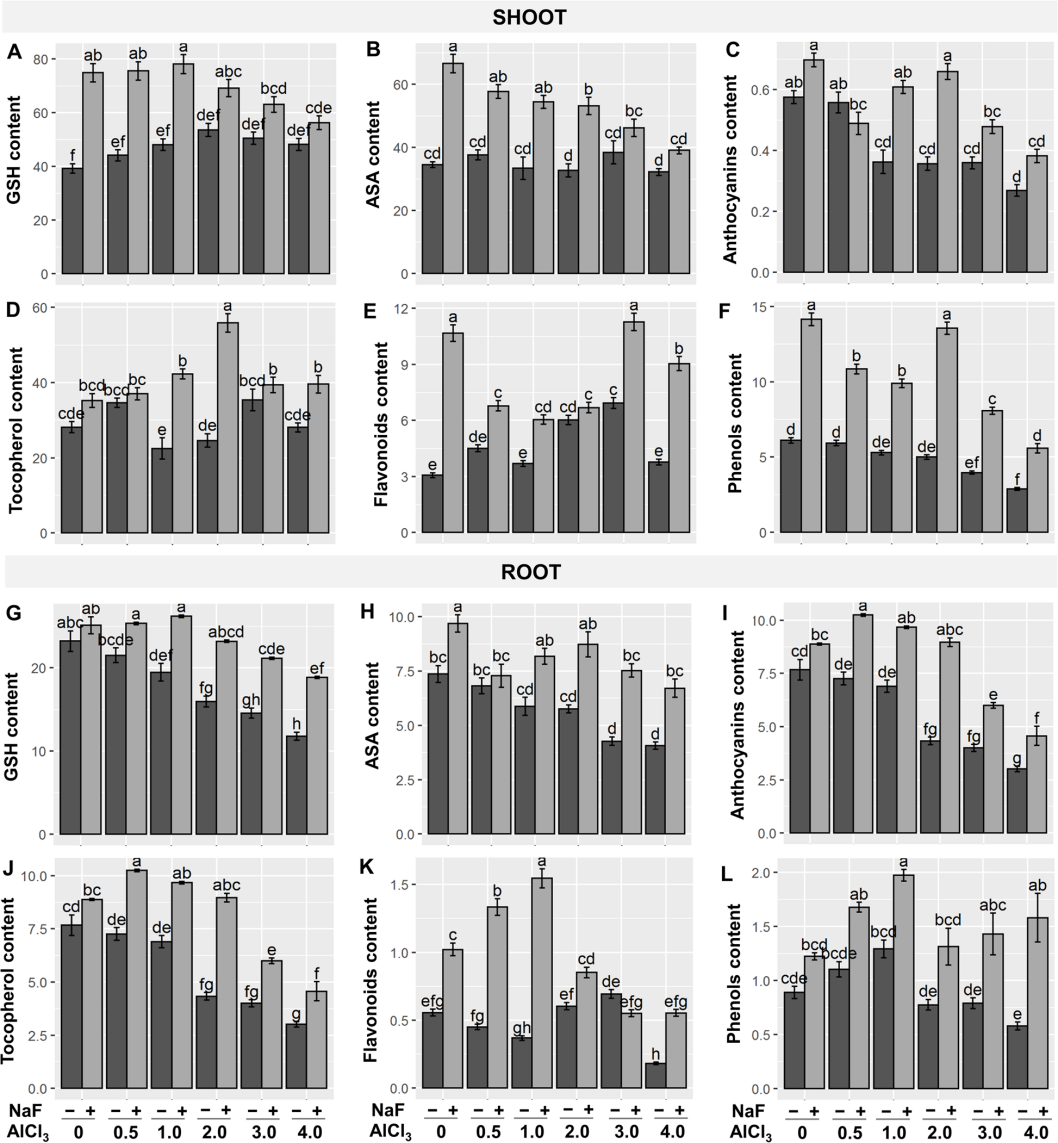
Effects of NaF priming on glutathione (GSH) (**A**, **G**), ascorbic acid (ASA) (**B**, **H**), anthocyanins (**C**, **I**), α-tocopherol (**D**, **J**), flavonoids (**E**, **K**), and phenolic compound contents (**F**, **L**) in shoots and roots of barley plants grown under Al-stress. Values are means ± standard errors (SEs) (*n* = 5). According to Tukey's test, bars that are accompanied by the same alphabet are not significant among the treatments at *P*≤0.05

Heavy metals are chelated intracellularly by GSH, ASC, flavonoids, and certain ligands such as metallothioneins (MC) and phytochelatins (PC) to eliminate excessive buildup in plant cytosol [35]. Furthermore, non-enzymatic antioxidants can detoxify ROS and reduce oxidative stress in stressful situations [36]. In this study, we identified non-enzymatic antioxidants as well as several associated enzymes. GSH content was dose-dependently suppressed by Al-stress at roots, but showed a varied response at shoots when compared to controls (Fig. 4A, G) When Al-stressed plants were compared to control plants, ASC levels decreased at the roots in a dose-dependent way but did not change at the shoots (Fig. 4B, H). However, exogenous NaF application increased GSH and ASC contents in shoots and roots of 'NaF+Al_1_', 'NaF+Al_2_', 'NaF+Al_3_', and 'NaF+Al_4_' treatments as compared to their respective only Al-stressed treatments (Fig. 4A, B, G, H). Exogenous NaF significantly increased flavonoids content in 'NaF+Al_1_', 'NaF+Al_2_', 'NaF+Al_3_', and 'NaF+Al_4_' treatments when relative to the respective only Al-exposed treatments at both shoots and roots (Fig. 4E, K).

Under Al-stress, both shoot and root-anthocyanin contents were lowered compared to control treatment, however exogenous NaF significantly boosted shoot and root-anthocyanin contents relative to their corresponding only Al-stressed treatments (Fig. 4C, I). Shoot α-tocopherol level declined significantly in a dose-dependent manner, although root α-tocopherol content showed a varied response when compared to control plants in Al-stressed plants (Fig. 4D, J). Ali et al. [37] found that α-tocopherol content increased considerably under cadmium stress conditions. Exogenous NaF application increased α-tocopherol concentrations in shoots and roots of 'NaF+Al_1_', 'NaF+Al_2_', 'NaF+Al_3_', and 'NaF+Al_4_' treatments compared to their respective only Al-stressed treatments (Fig. 4D, J). Under Al-stress conditions, the concentration of free phenolic compounds varied between roots and shoots, as found in high bush blueberry plants [26]. Exogenous NaF considerably increased the concentration of free phenolic compounds in both shoots and roots when matched to their corresponding only Al-stressed treatments (Fig. 4F, L).

### Effects of NaF on secondary metabolites and Al-detoxification under Al-stress

**Fig. 5:**
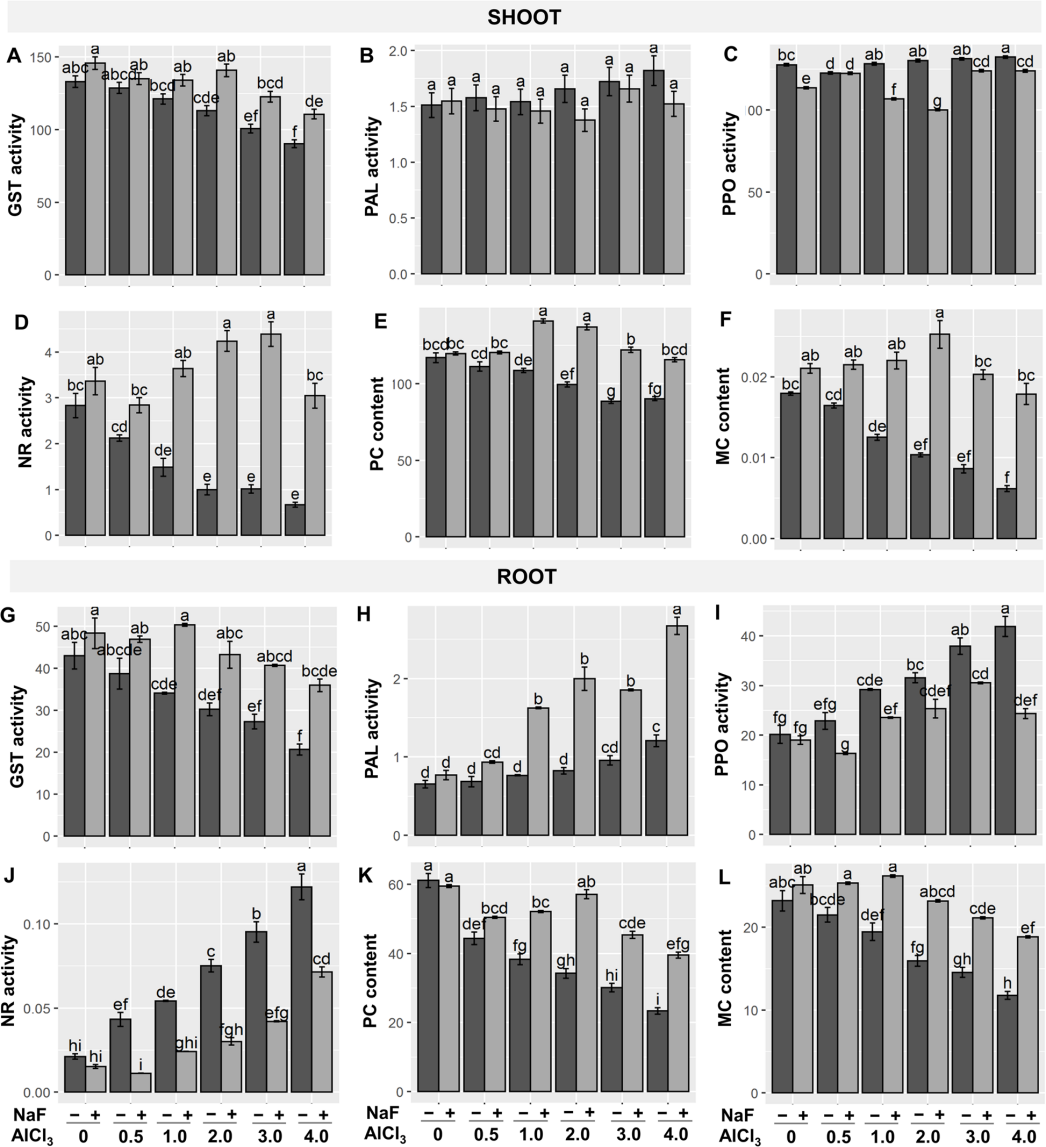
Effects of NaF priming on glutathione S-transferases (GST) (**A**, **G**), phenylalanine ammonia-lyase (PAL) (**B**, **H**), polyphenol oxidase (PPO) (**C**, **I**), nitrate reductase (NR) (**D**, **J**), phytochelatins (PC) (**E**, **K**), and metallothioneins (MC) contents (**F**, **L**) in shoots and roots of barley plants grown under Al-stress. Values are means ± standard errors (SEs) (*n* = 5). According to Tukey's test, bars that are accompanied by the same alphabet are not significant among the treatments at *P*≤0.05

When contrasted to the exclusively Al-stressed counterparts, Al-stress lowered glutathione-S-transferase (GST) activity in a dose-dependent manner at both shoots and roots, whereas exogenous NaF administration increased GST activity at both shoots and roots (Fig. 5A, G). Phenylalanine ammonia-lyase (PAL) activity revealed a varied response under Al-stress conditions, both at shoots and roots (Fig. 5B, H), while exogenous NaF treatment boosted PAL activity at roots but not at shoots compared to their respective only Al-stressed treatments (Fig. 5B, H). The activity of NR found to be reduced in shoots and increased in shoots in response to Al^+3^ stress **(**Fig. 5D, J). However, flouride application alleviated the reduction of shoots’ NR and attenuated their values in roots relative to the corresponding level. Furthermore, the activity of PPO **(**Fig. 5C, I) were increased in both organs, but highly significantly for roots, while the application of NaF decreased the activity of PPO shoots’ and roots. PC and MC contents were reduced in Al-stress treatments in a concentration-dependent way relative to control, but NaF priming significantly raised shoot-PC and root-PC and shoot-MC and root-MC contents relative to their corresponding only Al-stressed treatments (Fig. 5E, F, K, L). When compared to the control treatment, 'Al_2_' and 'Al_4_' treatments significantly boosted Al^3+^ uptake, however exogenous NaF lowered Al^3+^ concentrations in shoots and its uptake by roots relative to their respective solely Al-stressed treatments (Supplementary Fig. S1A, and B).

### Interactions between treatments and variables through heatmap and PCA

**Fig. 6:**
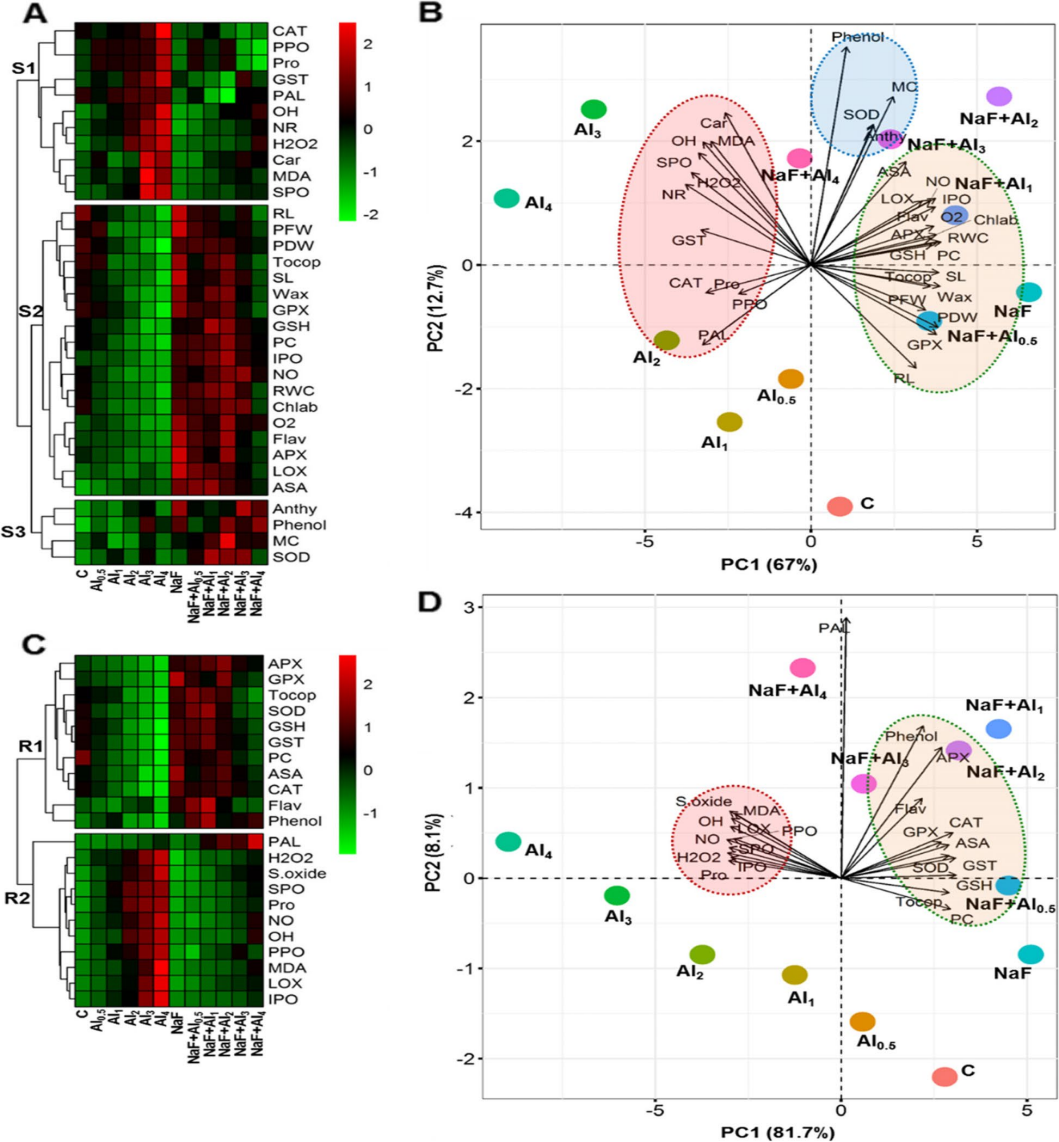
Hierarchical clustering with heatmap of studied parameters of shoot and root (**A**, **C**) and principal component analysis (PCA) of studied parameters of shoot and root (**B**, **D**) of barley plants. The variables included shoot length (SL), root length (RL), plant fresh weight (PFW), plant dry weight (PDW), epicuticular wax (wax), chlorophyll (chl), carotenoid (Car), proline (Pro), superoxide anion (S.oxide; O_2_^•−^), hydroxyl radical (OH), hydrogen peroxide (H_2_O_2_), malondialdehyde (MDA), lipoxygenases (LOX), and nitric oxide (NO), superoxide dismutase (SOD), catalase (CAT), soluble peroxidase (SPO), ionic peroxidase (IPO), ascorbate peroxidase (APX), glutathione peroxidase (GPX), glutathione (GSH), ascorbic acid (ASA), anthocyanins (anthy), α-tocopherol (tocop), flavonoids (flav), phenolic contents (phenol), glutathione S-transferases (GST), phenylalanine ammonia-lyase (PAL), polyphenol oxidase (PPO), nitrate reductase (NR), phytochelatins (PC), and metallothioneins (MC)

Hierarchical clustering divided all studied shoot-related parameters into three sub-categories (cluster-S1, -S2, and -S3) (Fig. 6A). Cluster-S1 contains CAT, PPO, Pro (proline), GST, PAL, ^•^OH, NR, H_2_O_2_, Car (carotenoids), MDA, and SPO parameters. Relative to control treatments, parameters of cluster-S1 displayed an increasing model in Al-treated barley plants, in the majority of cases, while those parameters exhibited decreasing model in NaF-primmed Al-stressed plants (Fig. 6A, C). However, RL, PFW, PDW, tocop (α-tocopherol), SL, wax (epicuticular wax), GPX, GSH, PC, IPO, NO, RWC, Chl ab (Chl *a*+*b*), s.oxide (O_2_^•−^), flav (flavonoids), APX, LOX, and ASA variables were grouped in cluster-S2. Cluster-S3 represented anthy (anthocyanin), phenol (free phenolic compounds), MC (metallothioneins), and SOD variables. Relative to control treatment, cluster-S2 and -S3 parameters exhibited a decreasing model in Al-exposed barley plants; on the other hand they showed an increasing model in NaF-primmed Al-stressed barley plants (Fig. 6A). In the case of root-related parameters, hierarchical clustering divided all studied parameters into two clusters (cluster-R1 and -R2). Cluster-R1 contains APX, GPX, tocop, SOD, GSH, GST, PC, ASA, CAT, flav, and phenol. Whereas, Cluster-R2 possesses PAL, H_2_O_2_, s.oxide (O_2_^•−^), SPO, Pro, NO, ^•^OH, PPO, MDA, LOX, and IPO (Fig. 6B). Relative to control treatment, cluster-R1 parameters displayed a decreasing model in Al-exposed barley plants whereas cluster-R2 parameters displayed an increasing model in Al-exposed barley plants. However, NaF application reversed the events in both of the clusters when relative to their corresponding Al-exposed plants (Fig. 6B).

The PCA biplot determined the level of the relation among the treatments, variables and treatments-variables. For shoot-related parameters, in total 79.70% of the data variability was covered by the principal component 1 (PC1) and PC2 (Fig. 6B). Only Al-stressed treatments were moderately or strongly connected with the parameters of PCA cluster-S1. On the other hand, NaF-primmed Al-stressed treatments were relatively strongly associated with the parameters of PCA cluster-S2, and -S3 (Fig. 6B). For root-related parameters, in total 89.80% of the data variability was covered by the PC1 and PC2 (Fig. 6D). Only Al-stressed treatments were moderately or strongly connected with the parameters of PCA cluster-R1. On the other hand, NaF-primmed Al-stressed treatments were relatively strongly associated with the parameters of PCA cluster-R2 (Fig. 6D).

## Discussion

Excessive Al^3+^ in soil solution interfere plant growth by hampering physiological and metabolic processes [12, 38]. Previous research has uncovered that plants employ a variety of strategies to fight against Al-induced phytotoxicity, such as decreasing Al^3+^ uptake into the roots and transporting Al^3+^ to above-ground parts, improving the chelation and sequestration process, and boosting the antioxidant capacity of the plant [2, 15]. When plants are subjected to stress for a prolonged period, these protective mechanisms become overwhelmed, necessitating the assistance of an exogenous stimulator to activate and maintain the defense mechanisms. Numerous exogenous stimulants have been investigated to minimize the deleterious effects of Al-stress in diverse plant species [39, 40]. Nonetheless, some plant stimulators, such as NO and selenium, were phytotoxic in more significant quantities but protective at lower concentrations [41, 42]. Keeping this in mind, we investigated the efficacy of exogenous fluoride application in relieving Al-toxicity in barley plants for the first time. Fluoride is hazardous to both plants and animals at higher concentrations [43, 44]; however, in this study, we found that the application of a lower concentration of fluoride (0.025% NaF) can reduce Al-induced growth inhibition and oxidative damage, confirming the role of fluoride in reducing Al-toxicity in plants.

Root growth inhibition is a primary symptom of Al-toxicity [26], as also found in this study (Table 1). Al^3+^ accumulates in the root cell wall and binds to negative cell wall charges [45], resulting in the impairment of the root elongation process due to the inhibition of cell division [11]. As a result, the root becomes stunted and brittle, the root hair develops poorly, and the root tip becomes inflamed and injured [38]. Furthermore, increased uptake of Al^3+^ by roots (Supplementary Fig. S1B) aids in subsequent transport of Al^3+^ to the shoot and leaves, resulting in increased shoot Al^3+^ content (Supplementary Fig. S1A), which compromises overall plant growth and development (Table 1). However, NaF effectively inhibited Al^3+^ uptake in the root and lowered the content of Al^3+^ in the shoot (Supplemental Fig. 1A, B), implying that lower Al^3+^ absorption is associated with higher root and shoot growth and biomass, resulting in an overall favorable influence on plant growth and development (Table 1). Previously, Yang and his co-workers reported that the amount of free Al^3+^ declined with the elevation of F^−^ concentration in a nutrient solution. Moreover, they also detected an Al-F complex in the leaf cell sap from the plants treated with both F^−^ and Al^3+^ [22]. Thus, we speculate that a low dose of fluoride might produce an Al-F complex in plants that blocks the toxic property of Al^3+^.

Al-toxicity led to oxidative stress (Fig. 2G-I) and membrane lipid oxidation (Fig. 2J), leading to root injury and impaired root growth (Table 1, Fig. 1). Root injury in plants causes inadequate nutrition and water uptake [46]. For instance, leaf RWC was significantly reduced in Al-stressed barley plants in this study (Table 1). Moreover, increased proline buildup at roots and shoots suggested physiological water limitation in barley plants (Table 1). Proline can protect proteins from stress by acting as an osmoprotectant [47]. As a result, the accumulation of proline content in plants during stress conditions is regarded as an osmotic stress signal [47]. However, NaF treatment increased RWC in Al-stressed barley plants, resulting in a drop in proline levels in the root (Table 1), indicating that exogenous NaF aids barley plants in combating Al-induced osmotic stress.

Photosynthetic pigments, notably Chl, indicate the status of plant health; thus, Chl content is a potential indicator of stress tolerance [33]. A rise in Al^3+^ levels in shoots resulted in a decrease in Chl *a*+*b* content (Table 1). Higher Al^3+^ concentrations in nutrient solutions may disrupt the absorption and transportation of some essential nutrients, such as Mg^2+^ [15], an indispensable mineral content for Chl biosynthesis [48]. Higher concentrations of Al^3+^ also increase the activity of Chl-degrading enzymes [49], resulting in decreased Chl *a*+*b* content (Table 1). However, NaF application enhanced Chl *a*+*b* content in Al-stressed barley plants more than only Al-stressed treatments (Table 1). Thus, improved preservation of photosynthetic pigments by administrating NaF may improve plant growth under Al-stress. It was also confirmed by PCA, where a strong association of Chls with NaF-treated Al-stressed treatments was observed (Fig. 6B, D).

Al-stress negatively affects metabolic processes in plants, particularly the balance between reactive species generation and detoxification [50]. MDA is a membrane lipid peroxidation product that implies severe membrane structural degradation. Under heavy metal stress, LOX activity rises, and a higher LOX activity is responsible for greater MDA production [15, 51]. Plant antioxidant systems include enzymatic (SOD, CAT, SPO, IPO, APX, and GPX) as well as non-enzymatic (GSH, ASA, anthocyanin, carotenoids, α-tocopherol, flavonoids, and free phenolic compounds) that play an essential role in reducing oxidative stress [50, 51]. Moreover, both GSH and ASA are required as substrates to detoxify H_2_O_2_ through the activities of GPX and APX, respectively [50]. In our study, antioxidant components of barley plants were depressed in most cases when exposed to Al-stress (Figs. 3 and 4). As a result, a sharp increase in ROS (O_2_^•−^, ^•^OH, and H_2_O_2_) contents, LOX activity, and MDA content both in shoots and roots was observed (Fig. 2A-E), leading to the impairment of optimum growth and development of barley plants (Fig. 1, Table 1). However, our findings showed that NaF application in barley subjected to Al-stress significantly stimulated the antioxidant systems (Figs. 3 and 4), causing a sharp reduction of ROS content (O_2_^•−^, ^•^OH, and H_2_O_2_) and MDA content both in shoots and roots of barley plants (Fig. 2A-E) and protected plasma membrane of plants leading to improved growth and biomass (Table 1). These findings were highlighted by PCA, which revealed that NaF-treated Al-stressed plants, both at the shoots and roots, had a stronger and more positive association with most of the antioxidant components than only Al-stressed plants (Fig. 6B, D), indicating that NaF plays a role in reducing oxidative stress.

Moreover, PPO catalyzes the production of reactive *O*-quinones via the oxidation of monophenols and/or *O*-diphenols that subsequently form ROS via interaction with oxygen and proteins [52]. In this experiment, higher PPO activity in Al-stressed conditions was reversed under NaF application (Fig. 5C, I), which might relate to the NaF-induced protection against Al-induced stress. In plants, NO acts as a strong oxidant or an effective antioxidant, entirely relying on some factors such as the concentration and the status of the stress. Elevated NO content in plants' physiological systems is connected to possible damage to photosynthetic electron transport, the reticence of plant growth, DNA damage, and cell death [53, 54]. However, NO boosts the growth-related and developmental processes of rice plants at lower concentrations [42]. In our experiment, dose-dependent increment of both shoot- and root-NO contents was reported in barley plants exposed to Al-stress (Fig. 2F, L), suggesting that NO functioned as a potent oxidant which might subsequently function in disturbing the developmental process of barley (Fig. 1, Table 1). Studies revealed that NO production in plants could be achieved via two main routes: one is reductive, and the other is oxidative. Reductive pathways include both the enzymatic and non-enzymatic processes of nitrite [55]. In our experiment, it was obvious that plants subjected to Al-stress displayed increased NO content with a concomitant decrease in NR activity (Figs. 2F, L, 5D and 4J), suggesting that the source of NO was not from the enzymatic reduction pathway. However, exogenous application of NaF increased NR activity but decreased NO content (Figs. 2F, L, 5D and 4J). These results suggest that increased NR might relate to nitrogen assimilation and metabolism, not excessive NO synthesis [55].

Nevertheless, to counteract heavy metal stress, plants are equipped with another pivotal mechanism such as intracellular chelators of metal ions by emitting some molecules, for example, organic PCs and MCs [15, 56]. PCs make complexes with metal ions, which are further sequestered into the vacuoles, and the formation of PC-metal complexes requires GST enzymes [57]. Under Al-stress, lowered GST activity was observed in barley plants (Fig. 5A, G). Moreover, in this study, PCs and MCs content were decreased in a dose-dependent manner both at shoots and roots (Fig. 5E, F, K, L), resulting in excess Al^3+^ accumulation both at shoots and roots (Supplementary Fig. S1A, B). However, NaF application increased PC and MC content and GST activity in plants subjected to Al-stress (Fig. 5A, E, F, G, K, L). This result suggests that a significant lower Al^3+^ content at both shoots and roots might be conferred by the sequestration of Al^3+^, which is related to high PC and MC contents and GST activity in NaF-treated Al-stressed plants.

The PCA and heatmap of all studied parameters of shoots and roots revealed that the fluoride-mediated Al-stress mitigation mechanism works slightly differently in shoots and roots (Fig. 6). A strong and positive correlation between only Al-stressed treatments and ROS and lipid peroxidation in both roots and shoots (Fig. 6B, D) indicates that Al causes oxidative stress in both organs. However, PCA revealed a more substantial and positive correlation between non-enzymatic antioxidants and fluoride-treated Al-stressed treatments in the shoot, as well as a strong and positive correlation between both non-enzymatic antioxidants and enzymatic antioxidants and fluoride-treated Al-stressed treatments in the root (Fig. 6B, D). These results suggest that in shoots, non-enzymatic antioxidants are more critical than enzymatic antioxidants, while in roots, both enzymatic and non-enzymatic antioxidant systems play important roles in fighting against Al-induced oxidative burst. Furthermore, GST is highly correlated with fluoride-treated Al-stressed treatments in roots but not in shoots (Fig. 6B, D), indicating that GST-mediated Al^3+^ detoxification is highly functional in the roots of barley plants.

## Materials and methods

### Growth condition and experimental setup

A hydroponic experiment was performed under natural conditions of humidity, temperature, and light in the glasshouse. Uniform-sized barley *cv. Giza 143* caryopses (the caryopses of barely were purchased from Agriculture Research Center, Giza, Egypt) were disinfected using 1% sodium hypochlorite solution for 10 min. Then caryopses were incubated for 4 days at 20°C under dark conditions in a plastic tray filled with sawdust moistened with distilled water. Four-day-old barley seedlings were transplanted in Petri dishes (10 seedlings per dish) containing only nutrient solution or nutrient solution containing AlCl_3_ (Sigma, Aldrich) solutions (0.5, 1.0, 2.0, 3.0, and 4.0 mM, pH 4.0) with or without 0.025% sodium fluoride (NaF) (Sigma, Aldrich). The nutrient solution (mg L^−1^) composed of potassium nitrate (101.1), calcium nitrate (164.2), magnesium sulphate (48.2), monopotassium phosphate (23.0), ferric EDTA (3.7), boric acid (1.24), manganese (II) chloride (0.6), zinc sulfate (0.32), copper (II) sulfate (0.08), ammonium heptamolybdate (0.47) and the pH was adjusted using HCl (2mM). Five Petri dishes per treatment have been conducted. The concentration of NaF was selected based on a preliminary experiment where 0.025% NaF was the most effective concentration in enhancing germination and seedling growth of barley (data not shown). Different morpho-physiological measurements were performed after 15 days of germination, and samples were collected for biochemical parameter assessments.

### Growth responses, relative water, chlorophyll, carotenoid and proline content determination

Morphological attributes such as SL, RL, PFW, and PDW of barley plants were measured. RWC and epicuticular wax content of barley leaves were measured following the methods as described earlier by Tahjib-UI-Arif et al. [58] and Kakani et al. [59], respectively. Leaf pigments for example Chl *a*, Chl *b*, and carotenoid contents were estimated based on the method of Lichtenthaler and Wellburn [60]. Fresh leaves were suspended in ethanol (95%) overnight and the absorbances of extracted pigments were recorded at 663, 644, and 452 nm. Proline contents of leaves and roots were estimated using ninhydrin [61]. Briefly, samples were homogenized with 3% 5-sulfosalicylic acid and supernatants were collected. Equal amount of ninhydrin reagent and glacial acetic acid were mixed with supernatant and heated at 95°C for 45 min. After cooling, toluene was added and mixed thoroughly. The absorbance of the toluene fraction was recorded at 520 nm wavelength.

### Determination of ROS content, lipid peroxidation level, NO content, LOX and NR activity

H_2_O_2_, O_2_^•−^, and ^•^OH contents of barley leaves and roots were determined according to the proposed protocols [62–64]. H_2_O_2_ content was quantified by homogenizing leaves in cold acetone and the extract then was mixed with sulfuric acid-titanium dioxide reagent and the developed color was recorded at 420 nm. O_2_^•−^ content was evaluated by homogenizing fresh samples in potassium-phosphate (K-P) buffer. The supernatant was collected after centrifugation and mixed with hydroxylamine hydrochloride and naphthylamine, respectively. Finally, the chromophores’ optical density was recorded at 520 nm. The ^•^OH content was determined by suspending the fresh tissues in K-P buffer supplemented with 2-deoxy-D-ribose at 37°C for 2 h. The suspended solution was incubated in glacial acetic acid and thiobarbituric acid dissolved in sodium hydroxide and then boiling in water bath for 10 min. The absorbance was recorded at 532 nm.

Lipid peroxidation product MDA content [65] and LOX activity [66] were determined following the published procedure. The fresh plant samples were homogenized in trichloroacetic acid (TCA) and centrifuged at 11500×g for 10 min at 4°C. The supernatant was mixed with TCA containing thiobarbituric acid and then heating the mixture at 95°C. The optical density was monitored at 532 nm. A substrate of linoleic acid in K-P buffer was used for LOX activity where the enzyme activity was done following the increment of absorbance at 234 nm. Content of NO and activity of NR were examined following the published procedure [67, 68]. NO content was extracted from fresh tissues by using glacial acetic acid and then the supernatant was treated with Griess reagent and kept for 30 min at room temperature and after that, the absorbance was monitored at 560 nm. Nitrate reductase (NR) activity was measured by incubating fresh tissues with K-P buffer and potassium nitrate in dark for 1 h. Then sulfanilamide and 1-naphthyl-ethylene diamine dihydrochloride were mixed and the absorbance was measured at 542 nm.

### Non-enzymatic antioxidants and secondary metabolites determination

ASA and GSH contents of barley leaves and roots were estimated based on the procedures [69, 70], respectively. Fresh tissues were macerated with 5% TCA solution, followed by adding 10% TCA to the clear supernatant and diluted Folin-Ciocalteu reagent was added. Finally, the intensity of blue color was monitored at 760 nm wavelength. For GSH quantification, TCA was used to extract GSH from fresh tissues and the clear supernatant was mixed with Ellman’s reagent. The α-tocopherol contents in leaves and roots were estimated following the published procedure [71]. Fresh tissues were homogenized with chloroform, and after centrifugation, dipyridyl reagent was added to the supernatant and then ferric chloride was mixed and the color developed was monitored within 50s at 522 nm. Flavonoids and anthocyanin contents were estimated according to the method proposed by [72, 73], respectively. Methanolic extract of plant tissues was used for detection of flavonoids. The extract was mixed with NaOH for 5 min, followed by the addition of NaNO_2_ for 6 min, AlCl_3_ for 6 min, and the final volume was adjusted to 5 mL with distilled water. The intensity of the color was measured at 510 nm wavelength in a spectrophotometer. Anthocyanins content was determined by homogenizing fresh tissues in acidified methanol. The supernatant was stored in darkness for 5 h at 5°C and then anthocyanins content was quantified using spectrophotometer at 550 nm.

Free phenolic compounds content was measured following a standard method [74]. Methanolic extract of fresh plant sample was treated with Folin-Ciocalteu reagent and sodium carbonate solution. The absorbance was taken at 720 nm and a standard curve of gallic acid was used to express total free phenolic compounds. Phytochelatins content was calculated by deducting the total GSH contents from non-protein thiols as proposed earlier [75] which was obtained by mixing supernatant of plant samples grounded in sulfosalicylic acid with Ellman’s reaction mixture [76]. The estimation of metallothioneins protein was measured according to Cataldo et al. [77]. Metallothionein was extracted in mixture of sucrose, Tris-HCl buffer, and mercaptoethanol (homogenization buffer). Chilled ethanol:chloroform was mixed with the supernatant followed by adding three levels of cold ethanol and kept for 1 h at -20°C. Centrifugation of was performed to obtain metallothionein pellets which mixed with ethanol:chloroform:homogenization buffer and then the pellets were left to air dry and then re-suspended again in Tris-HCl and ethylenediaminetetraacetic acid (EDTA). The produced mixture was incubated with 5,5-dithiobisnitrobenzoic acid for 30 min at room temperature and the absorbance was recorded at 412 nm.

### Enzymatic antioxidants activity determination

Fresh plant samples were crushed in liquid nitrogen and then were mixed with extraction buffer. The extraction buffer was potassium phosphate buffer (pH 7.8) containing EDTA and polyvinylpyrrolidone (PVP). The plant extracts were centrifuged at 11,500×g for 30 min at 4°C. The activities of SOD (EC 1.15.1.1) [78], CAT (EC 1.11.1.6) [79], APX (EC 1.11.1.11) [80], GPX (EC 4.3.1.5) [81], SPO and IPO [82] and PPO (EC 1.10.3.1) [83] were analyzed using Unico UV-2100 spectrophotometer. SOD activity was quantified by mixing the previous extract with epinephrine using sodium carbonate buffer where the increase in absorbance was measured at 480 nm. CAT was detected by following H_2_O_2_ consumption in K-P buffer and the decrement of optical denisity was measured at 240 nm. The activity of APX was monitored by mixing enzyme extract with K-P buffer extract in presence of EDTA, H_2_O_2_, and ascorbate at 290 nm. GPX activity was monitored by mixing enzyme extract with GSH in ice bath for 30 min and then the centrifuged. The supernatant was mixed with Na_2_HPO_4_ and 5,5`-dithio-bis-2-nitrobenzoic acid where the absorbance was measured at 412 nm. PPO activity was measured monitoring purpurogallin synthesis at 495 nm in reaction medium of K-P buffer and catechol for 5 min at 25°C. Then diluted H_2_SO_4_ was applied to the last mixture to stop the reaction, and absorbance was recorded at 495 nm. The activity of PAL was done in a reaction mixture of enzyme extract, borate buffer, and phenylalanine and then stopping the reaction by HCl, where the reaction was monitored at 290 nm. The activities of SPO and IPO were determined by mixing enzyme extract in a reaction mixture of K-P buffer, H_2_O_2_, and guaiacol was measured at 470 nm.

### Aluminum and fluoride content determination

Aluminium uptake of roots was done by hematoxylin staining method where the intact barley roots were washed thoroughly by tap water for 10 min, followed by mixing with hematoxylin and 0.02% KIO_3_ in dark for 15 min. After washing in distilled water for 10 min, equal length root tips were immersed in HCl for 1 h and absorbance was done at 490 nm. Al^3+^ content in barley shoots was determined using the atomic mass spectrometer (Thermo scientific, ICAP6200) and the fluoride content in barley seedlings was determined using a multiparameter photometer.

### Statistical analysis

The dataset was used for the analysis of two-way analysis of variance and after that Tukey’s test was performed to assess the significant difference between treatments at 5% (*P*<0.05) level of probability. The heatmap containing hierarchical clustering was prepared using the package ‘pheatmap’ and the packages ‘ggplot2’, ‘factoextra’, and ‘FactoMineR’ were employed to perform principal component analysis (PCA) in R 4.1.2.

## Conclusion

Exogenous fluoride successfully mitigated Al-induced (0.5 to 4 mM AlCl_3_) phytotoxicity and improved growth and biomass production of barley plants in a hydroponic culture where fluoride and Al^3+^ were applied together in the nutrient solution. Our findings highlighted that fluoride mediated Al-stress tolerance in barley plants primarily by (i) reducing the uptake of Al^3+^ and translocation of Al^3+^ from roots to shoots, (ii) sequestering and detoxifying Al^3+^ by upregulating GST, PC, and MC contents, (iii) balancing plant water relations via organic solute regulation, (iv) diminishing oxidative damage through enhancing both enzymatic and non-enzymatic antioxidants, and (v) protecting photosynthetic pigments. Furthermore, we suggest a comprehensive study on the molecular level and field trials along with an economic feasibility analysis to clarify the role of fluoride priming in preventing Al-toxicity and minimizing crop losses.

## Supplementary Information

**Additional file 1.** Effects of NaF priming on Al3^+^ content at shoot (A), Al3^+^ content at root (B), F^−^ (Flouride) content at shoot (C) where the plants were grown under different AlCl_3_ concentrations.

## Data Availability

All data generated or analysed during this study are included in this published article and its supplementary information file.
