# Supplementary material for: Fluoride mitigates aluminum-toxicity in barley: morpho-physiological responses and biochemical mechanisms
Source: BMC Plant Biol. 2022 Jun 13;22:287. doi: 10.1186/s12870-022-03610-z (PMC9190151; doi:10.1186/s12870-022-03610-z)
Supplement: Supplementary file 1 — Additional file 1. Effects of NaF priming on Al3+ content at shoot (A), Al3+ content at root (B), F− (Flouride) content at shoot (C) where the plants were grown under different AlCl3 concentrations. [file 12870_2022_3610_MOESM1_ESM.docx]

**Supplementary file**


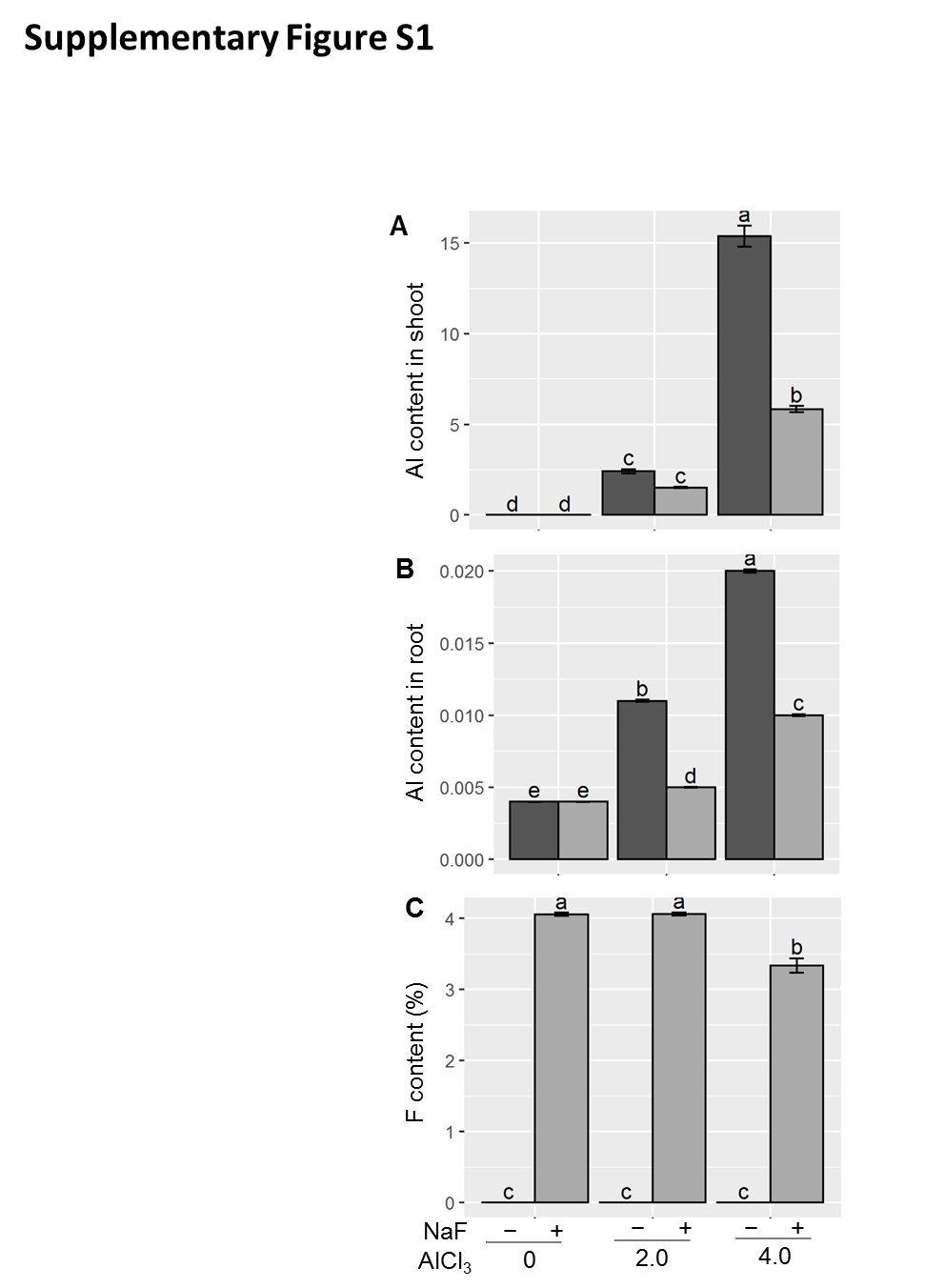


Al uptake

**Supplementary Fig. S1**

Effects of NaF priming on Al (aluminum content) at shoot (A), Al (aluminum uptake) by roots (B), F (fluorine content) of shoot (C). Values are means ± standard errors (SEs) (*n* = 5). Bars followed by the same letter are non-significant among the treatments at *P*≤0.05 based on Tukey’s test.
